# Supplementary material for: Causal Relationship between Aging and Anorexia Nervosa: A White-Matter-Microstructure-Mediated Mendelian Randomization Analysis
Source: Biomedicines. 2024 Aug 16;12(8):1874. doi: 10.3390/biomedicines12081874 (PMC11351342; doi:10.3390/biomedicines12081874)
Supplement: Supplementary file 1 [file biomedicines-12-01874-s001.zip › Supplementary Figure/Figure S4/Singe SNP plot of MR analysis for TL to ubm-b-1665.pdf]

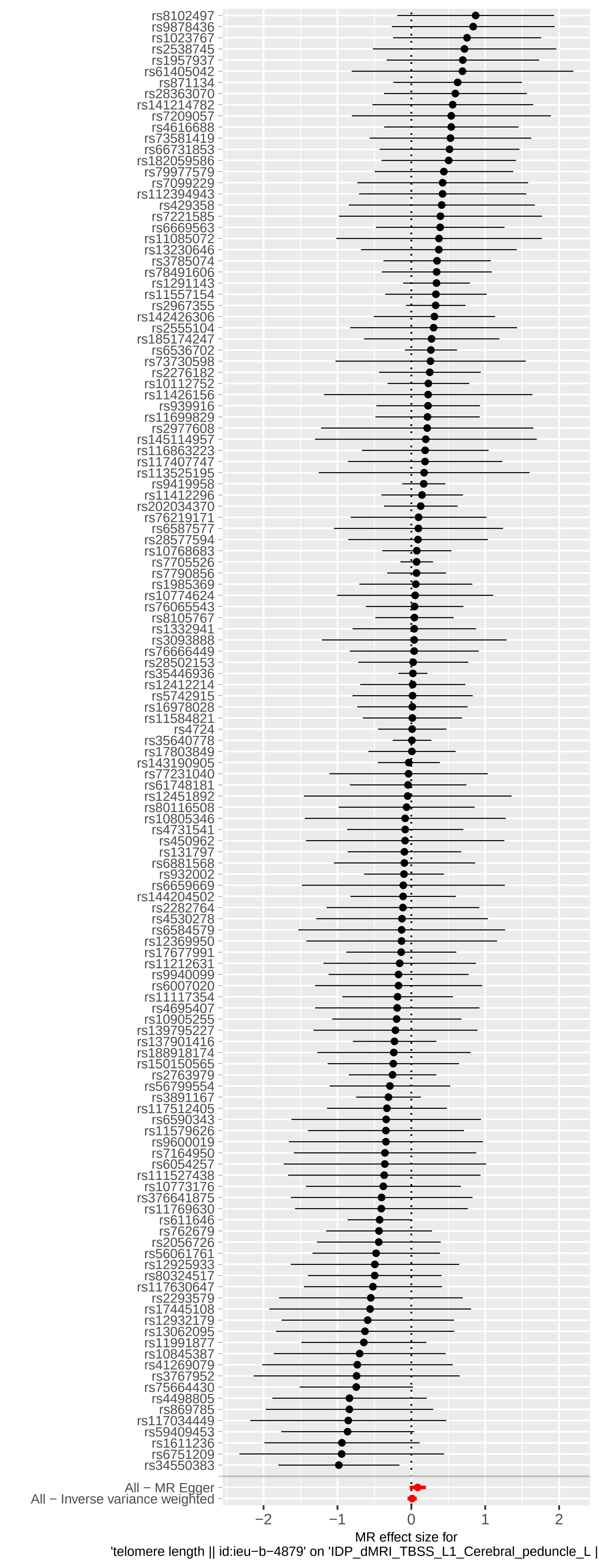
rs8102497
rs9878436
rs1023767
rs2538745
rs1957937
rs61405042
rs871134
rs28363070
rs141214782
rs7209057
rs4616688
rs73581419
rs66731853
rs182059586
rs79977579
rs7099229
rs112394943
rs429358
rs7221585
rs6669563
rs11085072
rs13230646
rs3785074
rs78491606
rs1291143
rs11557154
rs2967355
rs142426306
rs2555104
rs185174247
rs6536702
rs73730598
rs2276182
rs10112752
rs11426156
rs939916
rs11699829
rs2977608
rs145114957
rs116863223
rs117407747
rs113525195
rs9419958
rs11412296
rs202034370
rs76219171
rs6587577
rs28577594
rs10768683
rs7705526
rs7790856
rs1985369
rs10774624
rs76065543
rs8105767
rs1332941
rs3093888
rs76666449
rs28502153
rs35446936
rs12412214
rs5742915
rs16978028
rs11584821
rs4724
rs35640778
rs17803849
rs143190905
rs77231040
rs61748181
rs12451892
rs80116508
rs10805346
rs4731541
rs450962
rs131797
rs6881568
rs932002
rs6659669
rs144204502
rs2282764
rs4530278
rs6584579
rs12369950
rs17677991
rs11212631
rs9940099
rs6007020
rs11117354
rs4695407
rs10905255
rs139795227
rs137901416
rs188918174
rs150150565
rs2763979
rs56799554
rs3891167
rs117512405
rs6590343
rs11579626
rs9600019
rs7164950
rs6054257
rs111527438
rs10773176
rs376641875
rs11769630
rs611646
rs762679
rs2056726
rs56061761
rs12925933
rs80324517
rs117630647
rs2293579
rs17445108
rs12932179
rs13062095
rs11991877
rs10845387
rs41269079
rs3767952
rs75664430
rs4498805
rs869785
rs117034449
rs59409453
rs1611236
rs6751209
rs34550383
All − MR Egger
All − Inverse variance weighted
−2
−1
0
1
2
MR effect size for
'telomere length || id:ieu−b−4879' on 'IDP_dMRI_TBSS_L1_Cerebral_peduncle_L |
